# Pediatric Palliative Care in the Heart Failure, Ventricular Assist Device and Transplant Populations: Supporting Patients, Families and Their Clinical Teams

**DOI:** 10.3390/children8060468

**Published:** 2021-06-02

**Authors:** Kyle D. Hope, Priya N. Bhat, William J. Dreyer, Barbara A. Elias, Jaime L. Jump, Gina Santucci, Natasha S. Afonso, Margaret R. Ninemire, Barbara-Jo Achuff, Erin M. Kritz, Sharada H. Gowda, Kriti Puri

**Affiliations:** 1 Lillie Frank Abercrombie Section of Cardiology, Department of Pediatrics, Baylor College of Medicine, Texas Children’s Hospital, Houston, TX 77030, USA; kyle.hope@bcm.edu (K.D.H.); priya.bhat@bcm.edu (P.N.B.); wjdreyer@texaschildrens.org (W.J.D.); afonso@bcm.edu (N.S.A.); Rachel.Ninemire@bcm.edu (M.R.N.); Barbara-Jo.Achuff@bcm.edu (B.-J.A.); erin.kritz@bcm.edu (E.M.K.); 2 Section of Pediatric Critical Care Medicine, Department of Pediatrics, Baylor College of Medicine, Texas Children’s Hospital, Houston, TX 77030, USA; jaime.jump@bcm.edu; 3 Michael E. DeBakey Department of Surgery and Pediatrics, Baylor College of Medicine, Texas Children’s Hospital, Houston, TX 77030, USA; baelias@texaschildrens.org; 4 Section of Pediatric Palliative Care, Department of Pediatrics, Baylor College of Medicine, Texas Children’s Hospital, Houston, TX 77030, USA; gina.santucci@bcm.edu; 5 Section of Neonatology, Department of Pediatrics, Baylor College of Medicine, Texas Children’s Hospital, Houston, TX 77030, USA; sharada.gowda@bcm.edu

**Keywords:** palliative care, heart failure, ventricular assist device, heart transplant

## Abstract

Heart failure is a life-changing diagnosis for a child and their family. Pediatric patients with heart failure experience significant morbidity and frequent hospitalizations, and many require advanced therapies such as mechanical circulatory support and/or heart transplantation. Pediatric palliative care is an integral resource for the care of patients with heart failure along its continuum. This includes support during the grief of a new diagnosis in a child critically ill with decompensated heart failure, discussion of goals of care and the complexities of mechanical circulatory support, the pensive wait for heart transplantation, and symptom management and psychosocial support throughout the journey. In this article, we discuss the scope of pediatric palliative care in the realm of pediatric heart failure, ventricular assist device (VAD) support, and heart transplantation. We review the limited, albeit growing, literature in this field, with an added focus on difficult conversation and decision support surrounding re-transplantation, HF in young adults with congenital heart disease, the possibility of destination therapy VAD, and the grimmest decision of VAD de-activation.

## 1. Introduction

Heart failure (HF) is a growing cause of hospital admissions in children, with a significant morbidity and mortality burden [1,2]. Pediatric HF has been found to account for over 11,000 hospital admissions and over 5000 emergency room visits in the United States of America yearly [1,2]. The median hospital length of stay of a child with HF is nearly 20 days, with 12% having respiratory failure and over 5% having renal failure during the admission [1]. Additionally, a report from the Pediatric Health Information Systems (PHIS) database found that nearly 13% of patients admitted with HF were then re-admitted within 30 days after discharge, emphasizing the fragility of these patients [3]. Further, these patients have significant acuity, as recently illustrated by Lasa et al., who found that 6% of the admissions to cardiac intensive care units (ICUs) participating in the Pediatric Cardiac Critical Care Consortium (PC4) were patients in acute decompensated HF [4]. These patients had a hospital mortality rate of 15%, rendering pediatric acute decompensated HF to have one of the highest mortality rates in pediatric critical care medicine. Other studies on pediatric HF in the non-ICU setting have echoed this higher than baseline mortality, with an overall mortality rate of 5–7% seen in children admitted to the hospital with HF [1,2].

In the modern era, the survival of children with HF continues to improve with the increasing utilization of advanced therapies such as ventricular assist devices (VAD), extracorporeal membrane oxygenation (ECMO), and heart transplantation. Children diagnosed with HF have a 5-year transplant-free survival ranging from 30% to 60% depending on the etiology of HF, with those with congenital heart disease having lower survival [5,6]. A significant proportion of children diagnosed with HF will therefore go on to require advanced therapies such as a VAD, ECMO, or a heart transplant. Weighing the benefits of VAD support against the risk of its potentially serious complications represents an example of the complicated decision-making facing these patients and their families. Additionally, heart transplantation is also, at best, a palliation, as the median survival time of a transplanted heart is 12–20 years depending on the recipient’s age at transplantation [7]. Hence families and patients receiving a heart transplant may yet again face the challenges of worsening HF and decisions surrounding re-transplantation.

The aforementioned clinical scenarios, along with their morbidity and mortality, suggest the need for additional layers of support for a patient and/or family faced with the diagnosis of HF.

The field of pediatric palliative care (PPC) specializes in the symptomatic management of patients with serious illness, ensuring appropriate identification and pursuit of goals of care, and improving functionality and quality of life of patients and families living with serious illnesses [8,9,10]. The role of the PPC provider ranges from the emotional support of a patient and/or family during a medically complex admission or at the time of diagnosis of a serious life-altering illness, the management of symptoms (for e.g., pain, dyspnea, mood disturbances) during the course of illness, facilitating discussions about the quality of life and goals of care, and supporting families and care teams through end-of-life scenarios [8,9,10] A growing role of the PPC team is in supporting the care team of a patient with serious or life-limiting illness, including grief during emotionally challenging clinical scenarios, ethical dilemmas, as well as inter-disciplinary communication to help everyone be on the same page regarding the direction of care [9,10,11].

The American Academy of Pediatrics has previously recommended the development of pediatric palliative care (PPC) services to be an integral part of the multi-disciplinary care of children with terminal or life-threatening conditions [8]. Additionally, PPC is increasingly utilized in the care of critically ill children in the ICU [12,13,14]. Suggested criteria for palliative care involvement include an ICU length of stay longer than 2 weeks, respiratory failure with significant comorbidities, neurologic injury, or other serious or life-limiting conditions [12,13]. Specific to the field of pediatric cardiology, Wan et al. and Mazwi et al. have previously written about the role of the PPC team in the care of children with severe cardiac disease or critical congenital heart disease, including those on mechanical circulatory support or those being considered for heart transplantation [11,15]. Indeed the entire syndrome of HF, with its chronic life-altering nature, beckons for more involvement of PPC and its incorporation as a necessary and critical component of the care provided to these patients and families.

**Figure 1:**
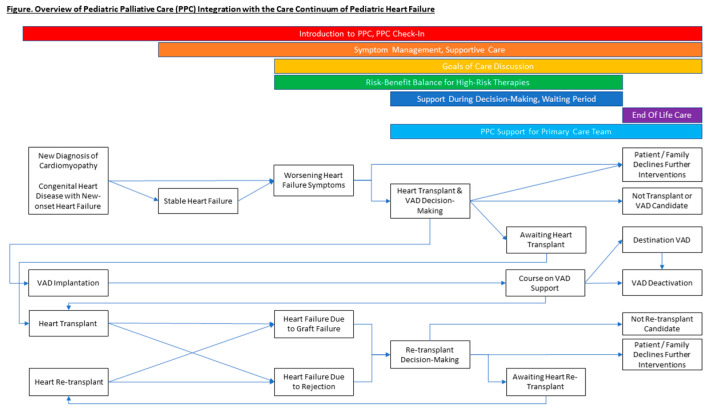
Pediatric palliative care throughout the continuum of pediatric heart failure and transplantation.

The goal of this article is to describe the avenues for incorporation of PPC in the management of a child diagnosed with HF, across the clinical trajectory from diagnosis through possible advanced therapies as well as end-of-life care (Figure 1). Written from the point of view of a multi-disciplinary team actively engaged in collaboratively caring for these patients, this article reviews the specific palliative care needs for each phase of the HF journey and hopes to serve as a roadmap for care teams looking to incorporate PPC in their practice.

## 2. Heart Failure

The diagnosis of HF is life-changing. It involves life-long medications, at times long-term infusions like milrinone administered through peripherally-inserted central catheters (with their own risks including catheter malfunctions and infectious complications), close monitoring and regulation of nutrition and fluid intake, possible need for nutrition repletion strategies such as gastric tubes due to the failure to thrive associated with HF, and the ever-looming possibility of worsening HF precipitating the need for heart transplantation or mechanical circulatory support. Children with HF are reported to have worse quality of life than their healthy peers as well as a worse quality of life when compared to children who have received a heart transplant [16,17]. While there are no reports on the routine utilization of PPC teams in the care of pediatric patients with HF, it may be beneficial to consider early PPC engagement if a child appears to be heading towards transplant or VAD. This enables early discussions and familiarity between the team and the family, which may be incredibly helpful if the patient ends up needing advanced heart failure therapies. Ideally, the role of the PPC team would evolve with the patient, adapting to their changing needs throughout the course of their HF. Visits could start with an introduction near the time of diagnosis, evolving to regular check-ins if the patient becomes hospitalized for HF management or mechanical support. PPC teams could furthermore assist with symptom management if end-organ function starts to deteriorate and support the patient and family through the waiting process while on the transplant list. This paradigm of longitudinal involvement of the palliative care team has also been discussed and reported in adults with HF [18] and may represent a useful framework to incorporating PPC in the care of children with HF.

## 3. Ventricular Assist Devices

Data on PPC utilization is more robust in the realm of children on VAD support. The field of pediatric VAD has grown in the last decade. Technological advancements, improvements in anticoagulation strategies, and better monitoring options, all leading to decreased complications, and the increase in a shared experience together contribute to better support for the sickest patients with HF [19]. However, while we are learning to maximize the support for patients who decades ago may have died without VAD support, this does not change the immensity of the decision for the parent and the care team, the catastrophic nature of complications such as strokes or major bleeding, and the fact that VAD support is not a permanent solution to HF. Knoll et al. reviewed their single-institution experience with PPC in children on VAD support and found that just over 40% received PPC consultations during the hospitalization, although the numbers were increasing with time throughout the study [20]. Of these, over half were done after the VAD was implanted. Additionally, in this study cohort, approximately a quarter of the patients died during their hospitalization. Further examination of these patients revealed that those receiving PPC consultation were more likely to have advanced directives in place and goal-concordant care regarding resuscitation efforts before their death. This suggests that PPC involvement may help facilitate earlier discussion about the goals of care and optimize end-of-life planning and care for these patients.

The management strategy behind VAD implantation also adds nuance to the role of the PPC team in the patient’s care. A patient who is supported on a VAD as a bridge to heart transplantation receives it as life-saving therapy, while also seeing a future transplant as the “light at the end of the tunnel”. However, even utilizing a VAD as a bridge to transplant is associated with stress to patients and their families. All younger children on paracorporeal VAD support (i.e., the Berlin Heart^TM^ (Berlin Heart, The Woodlands, TX)), and many older children with implantable continuous flow VADs (for example HeartWare HVAD^TM^ (Medtronic, Minneapolis, MN) and HeartMate 3^TM^ (Abbott, Abbott Park, IL, USA)) remain admitted to the hospital until the time of their transplant [19]. These prolonged lengths of stay are stressors for patients and families due to their financial, logistical, and emotional burden and the PPC team may provide consistent and longitudinal support during these long hospitalizations. Fortunately, the proportion of patients with implantable continuous flow VADs who are discharged home is increasing and is up to 55% in the most recent national registry reports [19,21]. However, 2/3 rds of these discharged patients have readmissions during their VAD course, with a median length of stay of 6 days. Further, children who are discharged from the hospital on VAD support must learn about their device, take regular medications including anticoagulants for which compliance is critical, and also still cope with many activity restrictions (e.g., no swimming, horseback riding, contact sports, or trampolining). Hence it is not surprising that children on VAD support have been found to have worse quality of life than healthy controls [22]. These aspects of chronic illness and technology dependence are additional features that make this population excellent candidates for additional support from a PPC team.

The second strategy of VAD implantation is as a bridge to decision. This is employed in patients who are either too sick to transplant (due to the severity of HF) or need more time to become better transplant candidates. Some examples of the latter include children with chemotherapy-induced cardiomyopathy who have not yet been in remission for a sufficient period of time to be listed for a heart transplant or children with chronic HF who need additional time for physical and nutritional rehabilitation. While supported on a VAD, these patients often remain admitted to the hospital, adding to their stress as described above. Staying motivated during rigorous therapy sessions is critically important but can be challenging during a long hospitalization and in the setting of chronic illness, requiring a driven family and patient. Additionally, there is a sense of uncertainty for the patient and the family: should the patient not be able to become a viable transplant candidate, all of this time on VAD support may “feel as if it is for nothing”. The support of the PPC team, in collaboration with the patient’s other teams, is key to keep the patient and family engaged during this period and provide assurance that they will be supported no matter what the outcome of the VAD support. This is also a clinical scenario wherein the PPC team can be a support for the primary care team of the patient, as providers for patients with lengthy and complex hospitalizations may face moral distress, especially if any morbidities are incurred along the way.

The final strategy of VAD implantation is VAD as destination therapy. This is an emerging concept as pediatric VAD management has become more sophisticated with fewer complications [23]. A destination VAD is the final cardiac palliation for a patient, at least for the foreseeable future, such as if the patient has an additional serious illness that will limit their life or render them a poor transplant candidate. Tunuguntla et al. illustrated a framework when approaching the question of whether a destination VAD is the right choice for a patient [23]. Critical to this assessment is the role of the PPC team to evaluate the goals of care of the family/patient and gauge their social and psychological abilities to care for a child on VAD support at home. Additionally, along with the primary care team, the PPC team can help the family understand the true burden of the VAD beyond the acute recovery from surgery to discharge, preparing them for possible readmissions for infectious or bleeding concerns, the intellectual burden of caring for a life-support machine 24-h-a-day at home, and symptom management for the patient if they are suffering from an additional illness such as chronic neuromuscular disease or terminal cancer.

A unique situation that may emerge for patients who are on VAD support as a bridge to a decision or as destination therapy is if there is a realization that VAD support no longer meets the goals that the patient/family wanted to achieve, and/or is not enough to change the eventual outcome. This may be due to a complication such as a devastating stroke or declining end-organ function that limits that their candidacy for a heart transplant, cancer recurrence with poor treatment prognosis, frequent readmissions that may have increased the level of morbidity and started to cause suffering, or an intercurrent illness that the VAD is not adequate to support the child through. These situations are some of the most gut-wrenching for care providers-the child is being kept alive by a machine but is that now still consistent with the family’s goals? The situation calls for PPC team support in numerous ways: addressing family goals of care, monitoring symptom management, discussing prognosis, supporting grieving families, facilitating conversations between various care providers, and support for the grieving members of the care team. The role of the PPC team in supporting the primary medical team in these situations cannot be overemphasized–they help manage any feelings of guilt or moral distress, offset the burden of managing all of the end-of-life care, and facilitate coping with provider grief through debriefing meetings following the patient’s demise.

In cases where VAD support is no longer consistent with the family’s goals of care, deactivation of a VAD can be performed. However, this clinical scenario remains a difficult situation for all involved. Kaufman et al. conducted a survey of providers for patients on VAD support to assess attitudes and knowledge about VAD deactivation [24]. Among the 106 respondents, two-thirds felt that they were not completely informed about the legal considerations around VAD deactivation. There was also little consensus about the indications for VAD deactivation, with only severe neurologic injury/stroke having an agreement of 88% of respondents, with a parent or patient request for deactivation being thought appropriate by just over 50% of those surveyed. Additionally, over 75% of the survey participants felt that it was reasonable to deactivate a VAD per parental request if the VAD support was prolonging suffering. This suggests that not only would patients and families benefit from PPC involvement when considering VAD deactivation, but that the teams could also use additional support and guidance at these times.

In this report, 84% of the respondents also felt that guidelines from a professional society regarding VAD deactivation in children would be beneficial [24]. Machado et al. published a VAD deactivation preparedness checklist for pediatric patients [25], which has been adapted from the adult checklist previously published by Schaefer et al. [26]. This document details the process of compassionate deactivation of mechanical circulatory support in four main stages. The first stage incorporates the identification of the various multi-disciplinary personnel who are key stakeholders in the process. After assessing their comfort and ability to be a part of the deactivation, there is a deep dive into the therapies that will need to be discontinued and the order in which to do so. The second stage identifies the key aspects of the family meeting, including its preparation, conduction, and documentation. This is the forum for the team to ensure that the family is on the same page regarding the various steps of the deactivation, as well as to learn about the family’s traditions, to enable incorporation (as best possible) in the deactivation process. This also allows appropriate medico-legal documentation regarding the advanced directives and decision-making responsibilities as well as the steps to be completed after deactivation. The third stage is to perform a “run-through” of the process and ensure that the providers and equipment/medications needed for the steps of deactivation are available and ready. The fourth stage describes the preparation of the family at the bedside, including ensuring the patient is comfortable as well as reviewing the family’s goals and the expected medical changes as the deactivation proceeds. This thorough checklist has been beneficial to us at our institution, in the case of 2 VAD deactivations in the hospital and 1 in a hospice setting. In our experience, some especially important aspects have been increasing the familiarity of the PPC team to VADs and their role in HF in general and reviewing the scenario and checklist in great detail. The emotion of the moment can be overwhelming for everyone involved, and, in hospice settings, having one of the primary cardiologists or PPC team members present to support the patient, their family, and the medical team members would be vital.

## 4. Heart Transplantation

The hope for all patients with end-stage heart failure is to survive successful heart transplantation. However, the timing is, naturally, unpredictable. Transplant waiting times can be long, especially in certain age groups such as infants, and can be longer for patients who have had multiple prior surgeries or have high levels of antibodies against potential donors (sensitization). Almond et al. reported the pediatric heart transplant waitlist mortality to be ~17% with only 63% of listed patients surviving to transplant [27]. Waiting for a heart transplant can be a stressful time for a parent, especially as it involves such conflicting emotions: for their child to live, another child has to die. Parents, and sometimes patients also, struggle with the dichotomy of this feeling, and the PPC team, in partnership with psychology, can be a vital support during this time. Additionally, if the waiting time is long, the severity of HF may worsen and the patient may become too sick to be able to survive a transplant surgery, thus negating their transplant candidacy. That precise scenario is why it is vital to have ongoing communication with the family throughout the waiting period so that if the clinical situation evolves, the discussion about goals of care can be carried out with a familiar team. Conversations regarding a patient no longer being a transplant candidate are difficult and often heart-wrenching for both families and providers. Having a PPC team that is well-known to all and actively involved early, from the time of transplant evaluation, may help in these difficult moments.

However, it is vital to recognize that heart transplant is not a cure-it is at best a palliation, and in the most practical sense, an exchanging of the current “serious illness” for another “serious illness” with its own unique medical concerns. It involves lifelong healthcare follow-up, medications that require strict compliance, may have cosmetic side-effects (such as weight gain with steroids and hirsutism with the immunosuppressant cyclosporine) as well as serious medical comorbidities (hypertension, diabetes, or kidney disease associated with calcineurin inhibitors like tacrolimus), some restrictions on diet and activities, and some limitations to the carefree attitude that every child desires. Add to that the annual biopsy procedures, potential admissions for rejection and infections, and the knowledge that the transplant does not last forever-all these represent the real medical burdens associated with heart transplantation facing patients and their families. Post-transplant, PPC teams may be more crucial than ever, as they help patients and their families navigate their changing environment and new unknowns ahead.

## 5. Heart Re-Transplant

As a heart transplant patient grows older, they may start showing evidence of graft dysfunction, which can occur secondary to a number of processes such as rejection and transplant-associated coronary artery disease. Depending on the specific clinical scenario, they may be able to undergo workup for a second heart transplant. However, if the first heart transplant failed within one year or if the patient has active transplant rejection on biopsy, they may potentially not be a re-transplant candidate. This makes their transplant graft failure a terminal condition. The patient and family may need support through the grief of this news as well as for deciding goals of care and/or limits of resuscitation. They may also need help with chronic symptom management including recurrent chest pain, a common symptom associated with worsening graft failure.

## 6. Adults with Congenital Heart Disease and Heart Failure—A Unique Population

A growing population of HF patients is that of adults with palliated or un-palliated congenital heart disease. These are young adults who underwent surgery in childhood for their structural heart disease which, while able to ensure survival to adulthood, still leaves them at risk for HF in the future. Burstein et al. found that the number of adult patients with congenital heart disease presenting to the emergency rooms with HF as well as those being admitted were increasing significantly over the past 10 years [28]. These patients are no strangers to the chronic nature of the illness—they have often been seen by cardiologists all their lives. However, now they are more often presenting to care for HF management. The etiology of their HF is often multifactorial: their palliated circulation may have a limited lifespan, complications such as arrhythmias and valvular dysfunction can accrue with time, and other organs such as the liver and kidneys can begin to fail due to multiple years of abnormal circulation. Hence, these patients often will see the diagnosis of HF as “yet another obstacle” in their long battle with heart disease. Due to their history of past surgeries, they may be more challenging transplant candidates due to complex anatomy or high levels of antibodies against possible donors. Furthermore, these patients have a higher mortality after VAD implantation or heart transplantation [29,30]. In light of the chronic nature of their disease, stressing the importance of good medical compliance, assessing the risk profile and prognosis of any additional surgical palliations, ensuring adequate psychological and social evaluation and management are all part of the recommended guidelines for their care [31]. The palliative care team, whether pediatric or adult, can be an incredibly helpful resource for this purpose. They can aid in engaging with these patients and examining their goals of care, assisting with symptom management or resource provision for mental health comorbidities, and helping them cope with the uncertain prognosis. Depending on their medical needs, these patients may transition care providers from pediatric to adult facilities. In this scenario, it is important to include palliative care among the medical specialties included in the transfer and ensure appropriate communication and “hand-off” to adult palliative care providers, to enable a smooth transition.

## 7. Conclusions

Pediatric HF is a life-changing and life-limiting condition, with significant morbidity and mortality. PPC is a critical part of the care for children with HF. Providing longitudinal support starting from the time of diagnosis, through repeat hospitalizations, counseling for a possible VAD or transplant, and the assessment of goals of care and management of psychosocial concerns are important roles of the PPC team. The support of the palliative care team may be especially beneficial for patients in families coping with uncertainty about heart transplantation or re-transplantation candidacy, those on VAD support as a bridge to a decision or as a destination therapy, and young adults with congenital heart disease and HF compounding their chronic healthcare needs. Early engagement of the PPC team will enable better communication with and support of the patient and family and allow timely discussions about important decision-making along the continuum of HF management.

## Data Availability

Not applicable.
